# A phase 0 study of the pharmacokinetics, biodistribution, and dosimetry of ^188^Re-liposome in patients with metastatic tumors

**DOI:** 10.1186/s13550-019-0509-6

**Published:** 2019-05-22

**Authors:** Shyh-Jen Wang, Wen-Sheng Huang, Chi-Mu Chuang, Chih-Hsien Chang, Te-Wei Lee, Gann Ting, Ming-Huang Chen, Peter Mu-Hsin Chang, Ta-Chung Chao, Hao-Wei Teng, Yee Chao, Yuh-Min Chen, Tzu-Ping Lin, Ya-Jen Chang, Su-Jung Chen, Yuan-Ruei Huang, Keng-Li Lan

**Affiliations:** 1 Department of Nuclear Medicine, Taipei Veterans General Hospital, Taipei, Taiwan; 2 Department of Obstetrics and Gynecology, Taipei Veterans General Hospital, Taipei, Taiwan; 3 School of Medicine, National Yang-Ming University, Taipei, Taiwan; 4 Institute of Nuclear Energy Research, Taoyuan, Taiwan; 5 Department of Oncology, Taipei Veterans General Hospital, No. 201, Sec. 2, Shih-Pai Rd, Pei-Tou District, Taipei City, Taiwan; 6 Department of Chest Medicine, Taipei Veterans General Hospital, Taipei, Taiwan; 7 Department of Urology, Taipei Veterans General Hospital, Taipei, Taiwan; 8 Institute of Traditional Medicine, School of Medicine, National Yang-Ming University, Taipei, Taiwan

**Keywords:** ^188^Re, Liposome, Biodistribution, Dosimetry; phase 0 exploratory IND

## Abstract

**Background:**

Liposomes are drug nano-carriers that are capable of targeting therapeutics to tumor sites because of enhanced permeability retention (EPR). In several preclinical studies with various tumor-bearing mice models, ^188^Re-liposome that has been developed by the Institute of Nuclear Energy Research (INER) demonstrates favorable in vivo tumor targeting, biodistribution, pharmacokinetics, and dosimetry. It inhibits the growth of tumors, increased survival, demonstrates good synergistic combination, and was safe to use.

This study conducts a phase 0 low-radioactivity clinical trial of nano-targeted radiotherapeutics ^188^Re-liposome to evaluate the effectiveness with which it targets tumors and the pharmacokinetics, biodistribution, dosimetry, and its safety in use. Twelve patients with metastatic cancers are studied in this trial. Serial whole-body scans and SPECT/CT are taken at 1, 4, 8, 24, 48, and 72 h after intravenous injection of 111 MBq of ^188^Re-liposome. The effectiveness with which tumors are targeted, the pharmacokinetics, biodistribution, dosimetry, and safety are evaluated using the VelocityAI and OLINDA/EXM software. Blood samples are collected at different time points for a pharmacokinetics study and a safety evaluation that involves monitoring changes in liver, renal, and hematological functions.

**Results:**

The T_½_z for ^188^Re-liposome in blood and plasma are 36.73 ± 14.00 h and 52.02 ± 45.21 h, respectively. The doses of radiation that are absorbed to vital organs such as the liver, spleen, lung, kidney, and bone marrow are 0.92 ± 0.35, 1.38 ± 1.81, 0.58 ± 0.28, 0.32 ± 0.09, and 0.06 ± 0.01 mGy/MBq, respectively, which is far less than the reference maximum tolerance dose after injection of ^188^Re-liposome. ^188^Re-liposome is absorbed by metastatic tumor lesions and the normal reticuloendothelial (RES) system. Certain patients exhibit a therapeutic response.

**Conclusion:**

This phase 0 exploratory IND study shows that nanocarrier ^188^Re-liposome achieves favorable tumor accumulation and tumor to normal organ uptake ratios for a subset of cancer patients. The clinical pharmacokinetic, biodistribution, and dosimetry results justify a further dose-escalating phase 1 clinical trial.

**Trial registration:**

Taiwan FDA MA1101G0 (Jan 31, 2012).

## Introduction

Cancer is a leading cause of death worldwide, and there is a crucial need for effective treatments [1]. Common flaws in many anti-cancer drugs include a lack of specificity, poor solubility and biodistribution, an unfavorable pharmacokinetic profile, and a propensity to induce tissue damage. Targeted drug delivery systems, such as self-assembled liposome, polymer, albumin, carbon nanotube, micelles, dendrimer, and hydrogel [2–7], have been developed to improve the poor bioavailability, unfavorable pharmacokinetics, multi-drug resistance, and tumor micro-environmental heterogeneous and systemic toxicity of anti-cancer drugs [8–11].

Nanoscale liposomes are widely used as drug delivery systems for chemotherapy drugs for the treatment of cancer. Many of the pharmacological properties of conventional chemotherapy drugs are improved by using this drug delivery system, which is composed primarily of lipids and/or polymers. These novel therapeutic complexes improve the pharmacokinetics (PK), biodistribution (BD), and safety (lower toxicity) of the coupled chemotherapeutic drugs. PEGylated liposome-coupled chemo-drugs have a longer circulating time than conventional chemotherapy. Liposome nanoparticles with sizes ranging from 100 to 200 nm extravasate from the blood flow into the tumor site and passively accumulate through leaky vasculature due to enhanced permeability and retention (EPR) [12], so radionuclides or chemotherapeutics are delivered to the tumor tissue sites [13, 14].

One of the most notable examples is PEGylated liposomal doxorubicin, which is approved for cancer treatment and is substantially less toxic than the doxorubicin free drug. Because of its high efficacy and low toxicity, the liposome-complex drug system is the most common nanomedicine that is approved by the FDA (Food and Drug Administration) for oncological indication [14, 15]. A study of the dosimetry of radionuclide-liposome in radiotherapy showed that radionuclide-liposome targeting therapy is feasible and normal vital tissue such as the liver and red marrow absorb radiation doses of ^188^Re-liposome (SUV; small unilamellar vesicles) of 0.44 mGy/MBq and 0.12 mGy/MBq, respectively. The tumor absorbs 0.405 mGy/MBq [16].

Internal radionuclide therapy delivers curative radiation to disseminated tumors. The effectiveness of radionuclide therapy depends on the tumor’s sensitivity to radioactivity and the amount of radiation that can be safely administered [17]. Radiosensitive tumors such as lymphomas respond well to radiation doses of 15–20 Gy, but solid tumors typically require 35–100 Gy for a meaningful response [17]. The radiosensitivity of normal bone also varies from bone marrow (typically > 1.5 Gy) to the lung and kidney (15–20 Gy). Rhenium-188 (^188^Re, *T*_1/2_ = 16.7 h) is a highly potent radionuclide that exhibits excellent physical and chemical properties as a nuclear radiotherapeutic [13, 18, 19]. Rhenium belongs to the manganese family (VIIB) as technetium-99 m and is widely used for labeling therapeutic radiopharmaceuticals. The therapeutic effect of ^188^Re derives from the E_β(max)_ value of 2.12 MeV (71.1%) which allows a maximum tissue penetration range R_β(max)_ of 10.4 mm and a linear energy transfer (LET) for 0.2 keV/μm beta radiation, but the 155-keV (15.6%) gamma ray with a half-life of 16.7 h allows tumor targeting to be monitored using gamma camera imaging [19]. However, the main disadvantage of ^188^Re radionuclide imaging is the presence of a high-energy background signal (478 keV 1.1%, 633 keV 1.4%, and 829 keV 0.4%).

A recent study used a high-energy ultra-high resolution multi-pinhole collimators to optimize both image quality and quantitative accuracy for ^188^Re SPECT imaging [20]. While new therapeutic radiopharmaceuticals are developing, the most significant advantage of ^188^Re over other radioisotopes is the availability and cost-effectiveness of the ^188^W/^188^Re-generator ((*T*_1/2_ = 69.4 days), which has a longer shelf-life and a higher specific activity. This is convenient to use and easier to continuously supply to remote areas of the world [19, 21]. A phase 1 clinical study showed a mean 3.60 GBq of ^188^Re-lipiodol with mean dose 4.5 Gy radiation absorbed in the liver for hepatocellular cancer treatment, which is an encouraging result [22]. One study of the treatment of hepatocellular carcinoma increased the activity dose of ^188^Re-lipodol from 4.8–7.0 GBq with a mean of 7.6 Gy to 15.2 Gy absorbed to the liver [23]. A therapeutic efficiency of 2.70–3.46 GBq Re-188-HEDP with a mean absorbed dose 11.8 Gy in bone metastases for bone pain palliation has also been reported [24].

Taking advantage of the EPR effect of nanoliposomal drug and the cytotoxic effect of β particles even in the absence of internalization of nanoliposome into the cancer cells, this study develops a nanoliposomal radiotherapeutic, ^188^Re-BMEDA-labeled PEGylated liposome (^188^Re-liposome) that effectively targets tumors and demonstrates good biodistribution, pharmacokinetics, and therapeutic effect in a tumor-bearing mice model [25–31]. Nano-targeted radiotherapeutics and micro-SPECT/CT imaging of ^188^Re-liposome shows a longer biological targeting half-life and better bioavailability, and the ^188^Re-liposome is more localized in tumor and ascites in mice that are inoculated with C26 colon carcinoma, either subcutaneously [25] or intraperitoneally [26].

Various tumor models consistently demonstrate the therapeutic and synergistic combination effects of ^188^Re-liposome with chemotherapeutics, including subcutaneously inoculated murine CT26 [28, 31], human LS-174 T [30], and a murine C26 colon carcinoma ascites model [27, 29]. Tumor growth is suppressed, survival time is increased [27, 29, 32], and there is a synergistic chemo/radiotherapeutic [28] effect in various tumor-bearing mice. The efficacy of ^188^Re-liposome as a nano-targeted radiotherapeutic has been demonstrated to be greater than that of the chemotherapeutics, Lipo-Dox [27, 28], 5-FU [29–31], and sorafenib [32]. Preclinical toxicity studies did not show any discernible toxicity in either mice [31] or rats [33, 34]. The encouraging data for the targeting of animal tumors, pharmacokinetics, biodistribution, dosimetric evaluation, preclinical efficacy, and toxicity resulted in a phase 0 low radioactivity human clinical trial to determine the potential of ^188^Re-liposome as a new passive nano-targeted anti-cancer radiotherapeutic. This study evaluated the pharmacokinetics, biodistribution, dosimetry, and tumor localization due to the EPR effect of nano-targeted ^188^Re-liposome in patients with metastatic cancer diseases.

## Methods

### Patient selection

An open-label, single-arm phase 0 trial was conducted at Taipei Veterans General Hospital (TVGH), Taipei, Taiwan, following the guidelines for exploratory IND study that were published by the US Food and Drug Administration (FDA) in 2006 [35] and approved by the Taiwanese FDA and the Institutional Review Board of the TVGH. All patients signed an informed consent document before participating in the study. The study was established to determine a safe starting dose for the phase I clinical trial and to obtain data for biodistribution, pharmacokinetics, radioactivity, and radiation dosimetry in patients who receive a single-intravenous bolus injection of ^188^Re-liposome.

The major criteria for inclusion in the study were as follows: Patients had to have a pathologically confirmed diagnosis of a primary solid tumor and pathologically or radiologically documented metastases that were refractory to current standard/available therapies. Patients had to have a Karnofsky Performance Status of at least 60% (or eastern cooperative oncology group (ECOG) performance status (PS) 0 to 2) and an anticipated survival of at least 3 months. Patients had to have recovered from any adverse events due to prior treatment (grade 0 or 1 in the common terminology criteria for adverse events (CTCAE) 4.03). Patients’ laboratory data had to show: absolute neutrophil count > 1.5 × 10^9^/L; platelet count > 100 × 10^9^/L; hemoglobin > 90 g/L; AST (aspartate aminotransferase), ALT (alanine aminotransferase), and ALP (alkaline phosphatase) ≤ 2.5 × ULN (upper limit of normal) (or ≤ 5 × ULN if known hepatic metastasis); total bilirubin ≤ 1.5 × ULN; and serum creatinine ≤ 1.5 × ULN (or calculated creatinine > 50 mL/min). Patients who had been subjected to any chemotherapy, radiation therapy, immunotherapy, angiogenesis inhibitors, and/or surgical treatment within 4 weeks preceding study entry were excluded.

Between Jul 2012 and Dec 2013, 12 patients with metastatic cancers that were refractory to currently standard or available therapies received an injection of 111 MBq of ^188^Re-liposome and then underwent imaging. Patients’ characteristics are summarized in Table 1. Fourteen patients received a ^188^Re-liposome injection with a mean administrated activity of 111 MBq. All patients tolerated the injection well, and there was no evidence of a significant infusion reaction, anaphylaxis, or signs of distress. Two of the 14 eligible patients withdrew from the trial because of abnormal liver function or intolerable pain from a metastatic mass: patient 05 withdrew consent on day 3 after the administration of ^188^Re-liposome due to deteriorating liver function and performance status. During a screening session, the ALT and ALP values for patient 05 were more than the ULN, which fulfilled the inclusion criteria for liver function for a patient with known hepatic metastasis (ALT and ALP ≤ 5 × ULN). However, a blood sample for patient 05 that was taken immediately before the injection of ^188^Re-liposome on day 1 showed an increase in AST and ALT to more than 200 U/L and a further increase to more than 800 U/L was recorded on day 2. Due to the patient’s deteriorating performance status and ongoing liver failure, the patient withdrew consent and did not undergo imaging on day 3 or day 4. The patient had existing multiple liver metastases due to lung cancer and there had been a rapid progression of hepatic failure before the injection so ^188^Re-liposome was not considered to be the cause of the deterioration in the condition of patient 05. Patient 06 withdrew consent after experiencing intolerable pain from a metastatic mass over the left axilla, extending to the upper back, while lying in a supine position during SPECT imaging. Therefore, data for dosimetry and biodistribution for these two patients is not included in the analysis. Two patients (patient 05 and 06) prematurely withdrew from the study without completing all of the imaging procedures, so 12 patients were included in the analysis of SPECT/CT imaging, pharmacokinetics, biodistribution, dosimetry, and safety of ^188^Re-liposome. Patient characteristics are listed in Table 1.

**Table 1:** Characteristics of the 12 patients

No.	Sex	Age	Primary cancer	TNM	Stage	Metastasis	Total %ID^a^	%ID/kg^b^
1	F	48	Breast	T2N2M2	IV	Bone	0.025	3.00
2	M	48	Esophagus	T1N0M1	IV	Pelvis	0.001	1.41
						Rectum	0.003	1.03
3	M	46	Liver	T3N0M1	IV	Bone	0.025	2.78
						Lung	0.004	3.56
						Liver)	2.580	9.03
4	F	61	Kidney	T4N0M1	IV	Bone	0.068	0.76
						Lung	0.001	3.22
7	F	58	Colon	T4N1M1	IV	Liver	0.017	10.12
8	F	46	Sarcoma	T2N0M1	IV	Lung	0.064	11.35
						Liver	0.018	7.36
9	M	66	Colon	T3N2M1	IV	Lung	0.083	3.71
10	M	63	Colon	T3N2M1	IV	Lung	0.012	2.10
11	M	64	Esophagus	T3N0M1	IV	Lung	0.061	3.99
12	M	80	Colon	T3N2M1	IV	Abdomen	0.029	1.37
						Bladder	0.040	1.10
						(Liver)	0.048	13.57
13	F	55	Colon	T4N1M1	IIIB	Lung	0.002	4.94
14	M	62	NPC	T2N1M1	IV	Lung	0.052	7.87

^a^Highest tumor uptake determined from ROI on 24-h SPECT imaging

^b^Percentage injected activity per kilogram calculated from estimated tumor volume

### Preparation of ^188^Re-liposomes

^188^Re was obtained from an alumina-based ^188^W/^188^Re generator. Elution of the ^188^W/^188^Re generator with normal saline yielded solutions of carrier-free ^188^Re as sodium perrhenate (NaReO_4_). A slightly modified form of the labeling procedure for ^188^Re-liposome that is described in [25 and 28] was used. The lipid compositions of pegylated liposomes contained hydrogen soybean phosphatidylchloine (HSPC), cholesterol, mPEG_2000_-DSPE (molar ratio 3:2:0.3), and ammonium sulfate solution with 250 mM (NH_4_)_2_SO_4_ (pH 5.0) in the inner water phase. Pegylated nanoliposomes have an average particle size of about 82.59 nm and contain 13.16 μmol/mL phospholipids. ^188^Re-liposome was manufactured in compliance with PIC/S GMP. BMEDA (ABX, Germany). Stannous chloride was used as the reductant, and sodium gluconate was used as an intermediate ligand to produce ^188^Re-SNS/S complexes. BMEDA (3.08 mg) was pipetted into a fresh glass vial, and sodium gluconate dissolved in 10% acetate solution was then added (0.34 mol/L). Stannous chloride was then added. When the solution had been flushed with N_2_ gas, ^188^Re-sodium perrhenate (0.9 mL) with a high specific activity (5.2~9.4 GBq/mL) was added. The vial was sealed and heated in an 80 °C dry bath for 1 h. The ^188^Re-BMEDA solution was adjusted to a pH of 6.0, prior to liposome (5 mL) labeling. The liposomes encapsulating (NH_4_)_2_SO_4_ were mixed and after-loaded with ^188^Re-BMEDA solution and incubated in a 60 °C dry bath for 30 min. A PD-10 column (G-25, GE Healthcare) chromatography with normal saline was used to separate radiolabeled liposomes from free ^188^Re-BMEDA, and a 0.22-μm filter was used to filter the solution. The ^188^Re-liposome injection was formulated with normal saline. The specification for the concentration of phospholipid was 3~6 μmol/mL, for particle size was 80~100 nm, for zeta potential was − 3~2 mV, and for radiochemical purity was more than 90%.

### SPECT/CT imaging and biodistribution

The dose of ^188^Re-liposome that was injected for this exploratory clinical study was determined based on previous animal studies [33] and the US FDA guidelines. In a study of ^188^Re-liposome toxicity in rats, the no adverse effect level (NOAEL) was 185 MBq per 200-g rat. The recommended starting dose for exploratory IND is 1/100 of NOAEL, so following the guidelines for exploratory IND study published by the US Food and Drug Administration (FDA) in 2006 [35], 111 MBq was selected for the low radioactivity phase 0 study. On day one, patients received an intravenous (i.v.) bolus injection of 111 MBq of ^188^Re-liposome and the time at which ^188^Re-liposome was administered was established as 0 h for the study. Information on biodistribution and dosimetry was derived from SPECT/CT data that was collected at 1, 8, 24, 48, and 72 h after injection with ^188^Re-liposome. Whole-body SPECT images were acquired over 60 min (1- and 8-h time points) or 90 min (24-, 48-, and 72-h time points) using a dual-headed SPECT/CT (Discovery™ NM/CT 670, GE Healthcare, Milwaukee, WS, USA) that was equipped with a medium-energy, general purpose collimator, for which the energy window was 155 + 10% KeV (139–170). Three bed positions (40 cm each) from head to thigh of SPECT/CT were performed for each time point set with a matrix size of 128 × 128 per image. The SPECT images were collected in step-and-shoot mode at 6° intervals over 180°. Sixteen-second projection views were obtained from each camera head. The images were reconstructed and analyzed using filtered back projection methods. Whole-body planar images were acquired at 8 cm/min from head to feet using the same machine. To identify the radioactivity level in tumors, major body organs, and muscle, volumes of interest (VOIs) were drawn based on CT images that were obtained within 24 h and merged with SPECT images using Velocity™ v2.8.1 (Velocity Medical Solutions, Atlanta, Georgia). VOIs for the muscle were placed over the left front thigh of each patient (Volume: 17.99 ± 2.66 ml). The VOI counts for normal and tumor areas at each time point were converted to a radioactivity level by multiplying factor 1 [36]. Factor 1 was calculated using the formula:

Factor 1 = *A*_1_/whole-body VOI counts

$$ {A}_1={A}_0\times {\mathrm{e}}^{-\left(\ 0.693/T1/2\right)1} $$

*A*_0_ and *A*_1_, respectively, refer to the radioactivity of the ^188^Re-liposome at injection time and the radioactivity at 1 h after injection. The results for biodistribution are presented as a percentage of the injected activity per kilogram (%IA/kg) for organs or tumors.

### Dosimetry

On the basis of the VOI counts for the whole body, major organs, and tumor obtained using Velocity™, the percentage of injected activity (%IA) for organs was calculated at various time points (1, 8, 24, 48, and 72 h after the injection of ^188^Re-liposome) and the %IA value for major organs was entered into the OLINDA/EXM [37, 38] software (Vanderbilt University, Nashville, TN, USA) to determine the dose that is absorbed by the brain, skin, bone, spleen, kidney, heart, liver, lung, intestine (large/small), bladder, muscle, stomach, testes (male only), ovaries (female only), and pancreas.

To determine the dose that is absorbed for different sizes of tumor, the %IA/kg value for the tumor, obtained at various time points (1, 8, 24, 48, and 72 h after injection with ^188^Re-liposome), was directly fitted with exponential models to calculate the number of disintegrations. This value was then input to the OLINDA/EXM computer program using the unit density sphere model [38].

### Safety monitoring

From days 1 to 4 and during follow-up visits on days 9 to 16 and 28 to 30 post-injection, the safety of ^188^Re-liposome was assessed by monitoring vital signs and checking for any indication of adverse events. Particular attention was given to infusion reaction, anaphylaxis, myelosuppression, or changes in hepatic or renal function. Blood samples were taken just before each SPECT scan at 1-, 8-, 24-, 48-, 72-h time points and at one extra time point (4 h) and during two follow-up visits for blood and plasma radioactivity analyses and hematological and biochemical studies. Urine samples were collected every 24 h to determine the daily and cumulative urinary excretion of ^188^Re-liposome. The severity of adverse events was graded using CTCAE v4.03. Laboratory tests (hematology, biochemistry, and urinalysis) were conducted during the screening visit, which occurred less than 10 days before ^188^Re-liposome was administered on day 1. These results were used as baseline data. Any adverse events (graded using CTCAE v4.03) or concomitant medications/therapies were recorded throughout the study using the case report forms (CRFs).

### Pharmacokinetics and urinalysis

Urine samples were collected every 24 h to determine the daily urinary excretion of ^188^Re-liposome. Radioactivity in urine was measured using a gamma counter at 24, 48, and 72 h after injection and the results are expressed as %IA/mL. In terms of pharmacokinetics, blood samples with anticoagulants were collected at 1, 4, 8, 24, 48, and 72 h and these were measured for radioactivity using the same gamma counter. The results are expressed as the percentage of injected activity per milliliter (%IA/ml). The pharmacokinetic parameters for ^188^Re-liposome in the blood were determined using WinNonlin software version 5.3 (Pharsight Corp., Mountain View, CA, USA). Non-compartmental analysis (NCA) was used to determine the pharmacokinetic parameters, including the terminal elimination half-life (*T*_1/2_z), the maximum concentration (*C*_max_), the total body clearance (Cl), and the area under the curve (AUC).

## Results

### Characteristics of ^188^Re-liposome

The characteristics of patients are listed in Table 1. The specification includes the concentrations of phospholipid as previously described. The radiochemical purity of ^188^Re-liposome was determined by running samples through PD-10 desalting columns containing Sephadex™ G-25 resin. The purity is 98.5 ± 1.3%, the mean specific activity is 63.2 ± 11.7 MBq/μmol (*n* = 12), the nanoparticle ^188^Re-liposome size range is 83.7 + 11.4, and the zeta potential of the nanoliposome is − 1.1 ± 0.7 mV.

### Pharmacokinetics and urinalysis

**Fig. 1:**
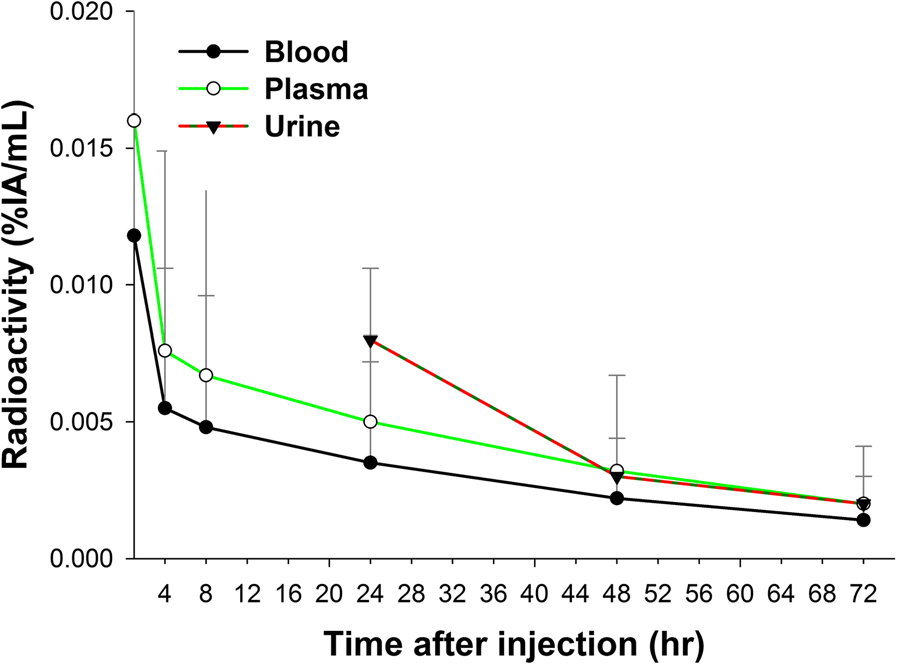
Blood, plasma, and urine clearance for nano-carrier ^188^Re-liposome in patients. The radioactivity that is measured in whole blood samples is identical to that in the plasma after centrifugation

The clearance of ^188^Re-liposome from the blood, plasma, and urine over the 72-h period of the study is shown in Fig. 1. Mean values for radioactivity in the blood and plasma of all patients decrease with time. At 1-h post-injection, the mean value for radioactivity is 0.012 ± 0.012%IA/ml in the blood and 0.017 ± 0.016%IA/ml in the plasma and this decreases to 0.002 ± 0.002%IA/ml in the blood and 0.002 ± 0.002%IA/ml in the plasma at day 4, post-injection. Urine samples were examined on days 2, 3, and 4, post-injection. The mean respective radioactivity values for the three examination times are 0.008 ± 0.005%IA/ml, 0.002 ± 0.001%IA/ml, and 0.002 ± 0.001%IA/ml, so the radioactivity level in urine also decreases over time, after injection.

The pharmacokinetic parameters are listed in Table 2. The maximum respective concentrations (*C*_max_) of ^188^Re-liposome in blood and plasma are 0.01 ± 0.01%IA/mL and 0.02 ± 0.02%IA/mL. The respective clearance rates (Cl) for ^188^Re-liposome from blood and plasma are 749.40 ± 663.19 mL/h and 494.26 ± 447.01 mL/h. The respective area under the time curve (AUC_0 → ∞_) for ^188^Re-liposome in blood and plasma is 0.33 ± 0.32% IA/g*h and 0.50 ± 0.46% IA/g*h. The respective values for *T*_1/2z_ (terminal elimination half-life) for ^188^Re-liposome in blood and plasma are 36.73 ± 14.00 h and 52.02 ± 45.21 h.

**Table 2:** Pharmacokinetic parameters for nano-carrier ^188^Re-liposome uptake after intravenous injection for patients (*n* = 12) with tumors

Parameters	Blood	Plasma
*C*_max_ (%ID/mL)	0.01 ± 0.01	0.02 ± 0.02
Cl (mL/h)	749.40 ± 663.19	494.26 ± 447.01
AUC_0→_∞(%ID /mL*h)	0.33 ± 0.32	0.50 ± 0.46
*T*_1/2Z_ (h)	36.73 ± 14.00	52.02 ± 45.21

Pharmacokinetic parameters are determined using WinNonlin software version 5.3 (Pharsight Corp., Mountain View, CA, USA)

*C*_*max*_ maximum concentration, *Cl* clearance rate, *AUC* area under curve, *T*_*1/2Z*_ elimination half-life

The urinalysis showed no clinically significant abnormalities that are related to the drug during the study period, with the exception of Patient 12, who showed a significant abnormality in the urine parameters, with significant proteinuria, hematuria, and leukocytes between day 2 post-injection and the last observation visit. In this case (Pt. 12), hematuria was only discovered at the screening visit and became clinically significant on day 2, post-injection. Although hematuria was identified as a SAE (serious adverse event), this adverse event appears to be unrelated to the study drug. No clinically significant abnormalities that are related to the ^188^Re-liposome injection are noted.

### SPECT imaging and the biodistribution of ^188^Re-liposome

**Fig. 2:**
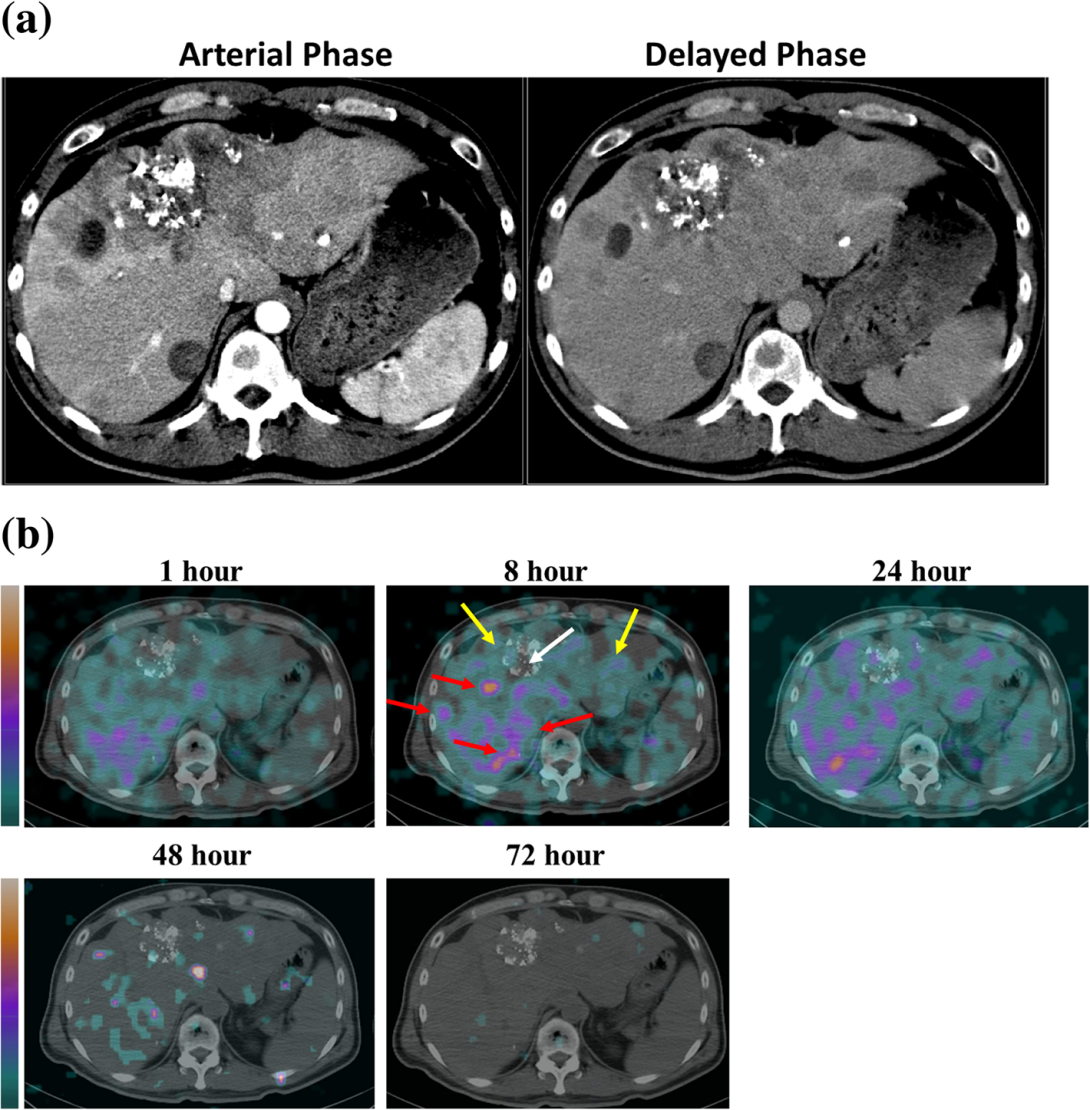
**a** Contrast CT image of a liver cancer patient (Pt. 3) with pulmonary and bone metastases displayed lipiodol retention resulted from transarterial embolization and multiple confluent nodular and mass lesions at both lobes of liver. **b** ^188^Re-liposome SPECT/CT imaging of tumor targeting and uptake in Pt. 3 at different time points after injection. The patient had previously received transarterial chemoembolization (TACE), which left a region with hyperdense lipoid as well as central necrotic area (white arrow). Several necrotic lesions show hypodense CT image and less radioactivity. Some viable tumor masses were either displayed as solid nodules or formed a rim-like structure surrounding the necrotic regions (red arrows). The radioactivity uptake of normal liver was indicated by yellow arrows

**Fig. 3:**
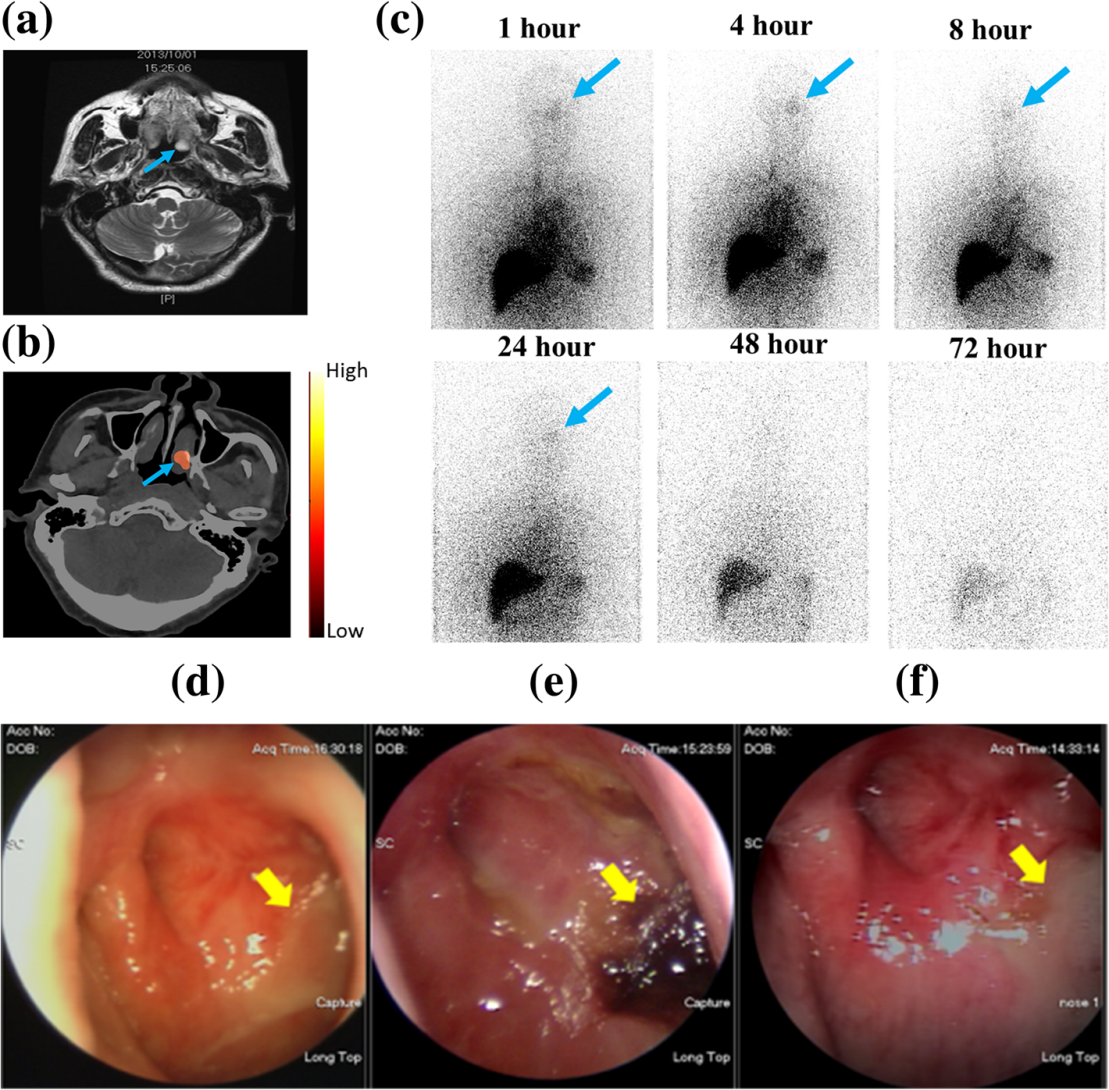
^188^Re-liposome SPECT/CT imaging showing tumor targeting and uptake in a NPC patient (Pt. 14) with pulmonary and mediastinal metastasis: **a** 1 month before ^188^Re-liposome injection, MRI there is a soft tissue lesion (blue arrow) in the left nasopharynx; **b** SPECT/CT 24 h after ^188^Re-liposome injection, there is a high uptake and efficient targeting (3.99%ID/kg) in the corresponding tumor lesion (blue arrow), as seen on the MRI; **c** anterior upper-body images for patient 14 at six different time points after ^188^Re-liposome injection (blue arrows indicate the lesion over the left nasopharynx); **d** the nasopharyncoscopic examination 1 month before ^188^Re-liposome injection shows the left nasopharyngeal mass with a crust and mucoid (yellow arrow); **e** 2 months after ^188^Re-liposome injection, there are engorged blood vessels and irregular surface over the left nasopharynx (yellow arrow); and **f** 1 year after the trial, the nasopharyngoscopic study shows fibrosis over the left nasopharynx (yellow arrow)

Figure 2 shows the transverse SPECT/CT images for patient 3, who exhibited hepatocellular carcinoma and multiple tumor masses over the liver, lung, and mediastinum. The patient had previously received transarterial chemoembolization (TACE), which left a region with hyperdense lipoid and a central necrotic area. Several necrotic lesions show as hypodense in the CT image and are less radioactive. Some viable tumor masses form a rim-like structure that surrounds the necrotic regions. Patient 14, who had a nasopharyngeal tumor (NPC) over the left nasal cavity, shows a high uptake and targeting (3.99%IA/kg) area at 24 h after injection with ^188^Re-liposome, which corresponds with the soft tissue lesion (blue arrow) that is seen on the corresponding MRI (Fig. 3a, b). Anterior planar images of patient 14 were obtained at different time points after injection with ^188^Re-liposome and show a discernible uptake of radioactivity in the left nasopharyngeal region (Fig. 3c).

The uptake of ^188^Re-liposome and tumor targeting measured using Velocity™ imaging software was used to calculate biodistribution of ^188^Re-liposome (%IA/kg) in normal organs for 12 patients from 1 h to 72 h. The results are listed in Table 3 for the spleen, kidney, heart, liver, lung, and muscle. The most prominent activity is noted in the spleen and liver at 1 h post-injection, but this decreases with time. The biodistribution of ^188^Re-liposome for the highest values for tumor targeting and uptake at each time point for 12 patients are summarized in Additional file 1: Table S1.

**Table 3:** Biodistribution for nano-carrier ^188^Re-liposome in normal organs of patients (*n* = 12)

Normal organ	Time point				
(%ID/kg)	1 h	8 h	24 h	48 h	72 h
Lungs	9.4 ± 2.3	7.5 ± 2.7	6.8 ± 2.6	3.7 ± 2.2	2.2 ± 1.4
Liver	14.7 ± 5.4	12.6 ± 4.9	11.2 ± 4.7	8.7 ± 4.4	5.9 ± 4.5
Heart contents	4.2 ± 1.9	3.1 ± 1.8	2.7 ± 1.4	1.4 ± 1.1	0.7 ± 0.5
Spleen	13.2 ± 8.0	12.3 ± 8.3	11.4 ± 7.3	9.0 ± 7.0	6.3 ± 6.3
Kidneys	8.1 ± 5.8	6.1 ± 2.5	5.7 ± 2.5	3.7 ± 1.5	2.1 ± 2.0
Muscle	0.4 ± 0.3	0.2 ± 0.3	0.1 ± 0.2	0.1 ± 0.1	0.1 ± 0.1

Data is expressed as a percentage of injected activity per kilogram (%ID/kg) ± SD

Normal organ assessment for the 12 subjects shows that the liver has the greatest biodistribution at 1 h post-injection (14.7 ± 5.4%IA/kg), followed by the spleen (13.2 ± 8.0%IA/kg) and lung (9.4 ± 2.3%IA/kg). Radioactivity in normal organs peaks at 1 h post-injection and then decreases slowly. The differences in tumor targeting and the uptake of radioactivity for the bone, pelvis, lung, liver, bladder, and NPC tumors are summarized in Additional file 1: Table S1.

### Dosimetry of ^188^Re-liposome

Using the biodistribution results for normal organs and tumors in Table 3 and Additional file 1: Table S1, the dosimetry of the absorbed radiation dose after intravenous injection of ^188^Re-liposome (111 MBq) was calculated. The results in Table 4 show that normal organs that absorb high doses include the spleen (1.38 ± 1.81 mGy/MBq), liver (0.92 ± 0.35 mGy/MBq), lungs (0.58 ± 0.28 mGy/MBq), red marrow (0.06 ± 0.01 mGy/MBq), and kidneys (0.32 ± 0.09 mGy/MBq). It is seen that the doses that are absorbed by these normal organs were much lower than the vital organ maximum tolerance doses.

**Table 4:** Absorbed doses (mGy/MBq) of ^188^Re-liposome in normal organs of patients (*n* = 12)

Normal organs	Absorbed doses (mGy/MBq)
Brain	0.08 ± 0.01
Small intestine	0.08 ± 0.01
Heart wall	0.22 ± 0.08
Kidneys	0.32 ± 0.09
Liver	0.92 ± 0.35
Lungs	0.58 ± 0.28
Red marrow	0.06 ± 0.01
Spleen	1.38 ± 1.81
Total body	0.20 ± 0.07
Effective dose	0.12 ± 0.02

Data is expressed as mean ± SD

The dose that is absorbed by tumor lesions because of the intravenous injection of ^188^Re-liposome (111 MBq) was then calculated. The highest doses of ^188^Re-liposome that are absorbed by tumor lesions for 12 patients are listed in Table 5. The highest dose that is absorbed in a liver tumor lesion is 0.86~1.70 mGy/MBq. Lung tumors (0.21~1.04 mGy/MBq) and bone tumors (0.12~0.28 mGy/MBq) also absorb higher doses than other tumor lesions.

**Table 5:** Highest absorbed doses (mGy/MBq) of ^188^Re-liposome in tumor lesions of patients (*n* = 12)

Tumor site	Absorbed dose (mGy/MBq)
Tumor in bone	
Pt. 1	0.28
Pt. 3	0.25
Pt. 4	0.12
Tumor in pelvis	
Pt. 2	0.16
Tumor in rectum	
Pt. 2	0.14
Tumor in lung	
Pt. 3	0.39
Pt. 4	0.29
Pt. 8	0.65
Pt. 9	0.33
Pt. 10	0.21
Pt. 11	0.32
Pt. 13	0.61
Pt. 14	1.04
Tumor in liver	
Pt. 3	0.86
Pt. 7	0.86
Pt. 8	0.90
Pt. 12	1.70
Tumor in bladder	
Pt. 12	0.16
Tumor in NP	
Pt. 14	0.15

*Pt.* patient

### Safety and clinical results

The results for complete blood count (CBC) and the biochemical data for blood are shown in Additional file 1: Figure S1 and Figure S2. The mean hemoglobin level, RBC and lymphocyte counts for the 12 subjects are within a normal or near-normal range throughout the study. No clinically significant abnormalities are seen. No serious adverse reaction is noted for any of the participants in the study.

**Fig. 4:**
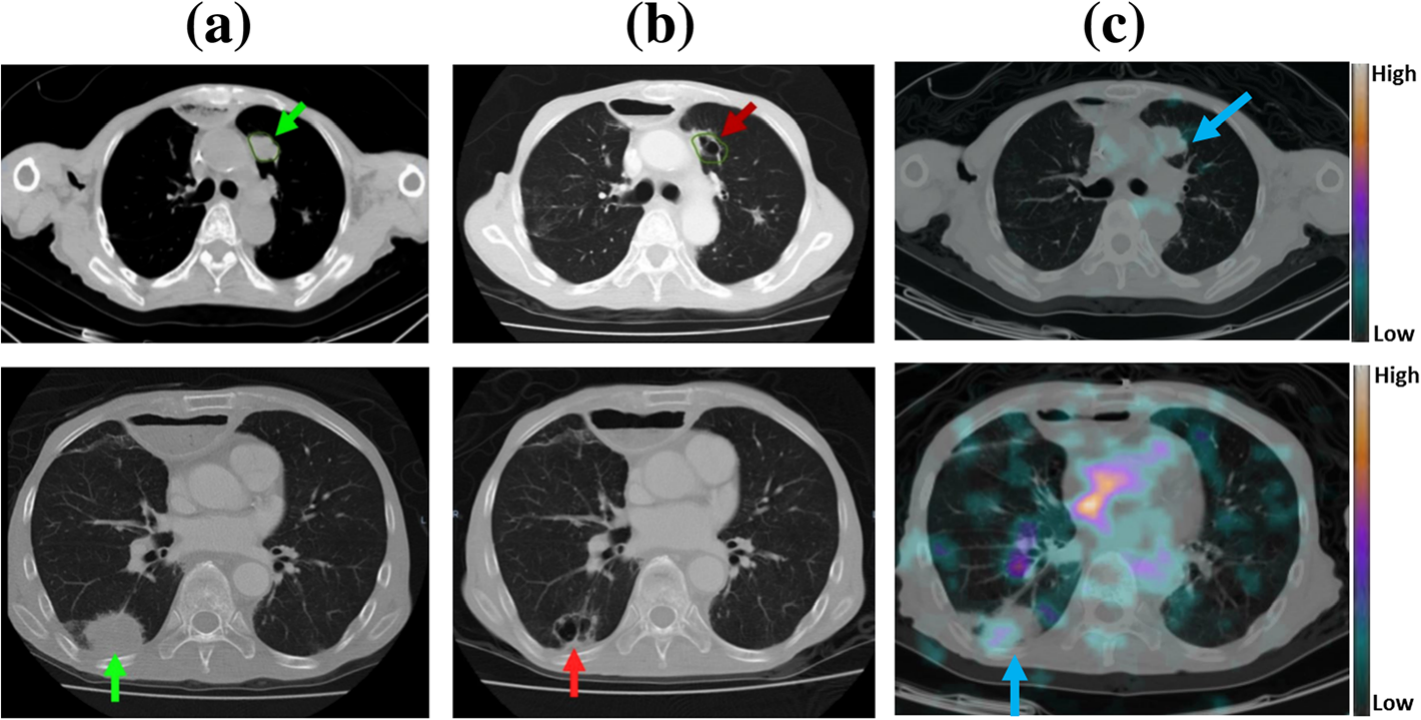
A series of chest CT images for patient 11, who had esophageal cancer and lung metastases tumors: **a** 1 month before and **b** 4 months after ^188^Re-liposome injection, the metastatic lesions (green arrows) either decrease in size or show signs of cavitation (red arrows) and **c** SPECT/CT images captured at 8 h after ^188^Re-liposome injection show a high uptake and efficient targeting in the corresponding tumor lesion (blue arrow), which is seen on CT

The exploratory study determines the effectiveness with which tumors are targeted and the pharmacokinetics, biodistribution, and dosimetry of ^188^Re-liposome but a study of patients’ subsequent imaging results and survival times (Table 1) shows that at least two patients exhibit a therapeutic response (Pt. 11 and Pt. 14). Patient 11 had esophageal cancer with progressive multiple lung metastases, as shown in Fig. 4. Within 4 months after injection with ^188^Re-liposome, during which time, the patient received no other therapy, and the follow-up CT sows cavitation of the metastatic lung lesions. Patient 14 experienced NPC with multiple lung metastases. The results of the nasopharyngoscopic examination show a NPC image with engorged blood vessels and irregular surface prior to the injection of ^188^Re-liposome, but significant ^188^Re-liposome uptake in the corresponding site (Fig. 3a, b).

## Discussion

The results show good tumor targeting, pharmacokinetics, biodistribution, and dosimetry for the intravenous administration of 111 MBq ^188^Re-liposome in a subset of 12 cancer patients who had exhausted all available therapies for their metastatic cancer. An advantage of ^188^Re-liposome over chemotherapy is that the delivery of a radiation-absorbed dose of internal nano-targeted radiotherapeutic to tumor sites does not require the release of the encapsulated radionuclide from the liposome. The advantages of ^188^Re for palliative radiotherapy are a short physical half-life of 16.9 h, a high-energy tissue penetration range of 2.1 MeV (R_β(max)_ 10.4 mm), and LET of 0.2 keV/μm for β particles. The 155 keV (15.1%) γ-rays also allow nuclear imaging. Patients rarely require hospitalization because the gamma irradiation rate is low and has a relatively short half-life, so this is an ideal radionuclide for clinical theranostic purposes. According to the U.S. Nuclear Regulatory Committee guidelines (Regulatory Guide 8.39-Release of patients administered radioactive materials), up to 29 GBq (790 mCi) of ^188^Re can be administered to outpatients. ^188^Re has been the subject of clinical research for the treatment of liver cancer [39] and prostate cancer with osseous metastases [40]. In these studies, ^188^Re was labeled on lipiodol to treat hepatocellular carcinoma [22, 23] or labeled on hydroxyethylidene diphosphonate (HEDP) for the palliation of metastatic bone pain [24].

The preclinical and validation animal research for this study shows that the use of nano-targeted ^188^Re-liposome prolongs exposure to the radiotherapeutic, and tumor tissue targeting and localization is better because the slower metabolism gives enhanced permeability and retention (EPR) [25–32]. Table 1 lists the characteristics of the 12 patients for this study. Figure 1 and Table 2 show the results of the pharmacokinetic study for the 12 patients. The respective terminal elimination half-life (*T*_1/2z_) of ^188^Re-liposome in blood and plasma is 36.73 ± 14.00 h and 52.02 ± 45.21 h. Other clinical trials obtained similar results [41–44]. Golan et al. [43], Chang et al. [42], Harrington et al. [41], and Giovinazzo et al. [44] reported that the respective *T*_1/2_ values for passive nano-targeted PEGylated liposomal mitomycin C prodrug, liposomal irinotecan, ^111^In-DTPA-liposomes, and PEGylated liposomal doxorubicin are 18~27 h, 58~75 h, 79.6 h, and 40~100 h. The prolonged circulation and increased biological half-life of ^188^Re-liposome allows the accumulation of Re-188 that is encapsulated in passive nano-targeted liposome in tumor sites because of EPR.

Table 3 summarizes the biodistribution of ^188^Re-liposome in normal organs for the 12 patients in this study from 1 h to 72 h. The liver and spleen exhibit the highest uptake. The results of the pharmacokinetic study show the biodistribution of nano-carrier ^188^Re-liposome in the tumors of patients (*n* = 12). There is uptake by the tumor and accumulation because of EPR (Additional file 1: Table S1). Figure 3a and b show the MRI image for a nasopharyngeal primary tumor (Fig. 3a) and the SPECT/CT with transverse imaging showing the uptake, accumulation, and targeting for ^188^Re-liposome (Fig. 3b) for NPC patient 14. The tumor uptake is 3.99%IA/kg at 24 h.

Although the biodistribution study demonstrates that ^188^Re-liposomes accumulate in solid tumors and remain there for prolonged periods, there is a significant difference in liposome uptake for different tumor types and for different patients with the same tumor type (Additional file 1: Table S1). There is a significantly lower uptake in bone tumors than in lung and liver tumors (Table 5). Tumors and normal organs are visualized using CT images and SPECT images that are merged using Velocity™ v2.8.1. The respective doses that are absorbed by tumors in the liver and lung are 1.08 ± 0.41 and 0.49 ± 0.29 mGy/MBq. These doses are higher than those for tumors within the bone, pelvis, rectum, bladder, and nasopharynx, which absorb doses of 0.12 to 0.28 mGy/MBq (Table 5). However, the normal tissues over the liver and lung also absorb relatively higher respective doses of 0.92 ± 0.35 and 0.58 ± 0.28 mGy/MBq (Table 4), which are similar to the uptake of radioactivity by tumors within these two organs. The tumors within the liver and lung have higher levels of radioactivity uptake, but this is not clearly shown by SPECT imaging due to the high background in these two normal organs. These results might be due to higher background uptake of the reticuloendothelial system (RES), accumulation in the abdominal and thoracic cavity, and interference in SPECT imaging due to a high-energy background signal [20].

The uptake (3.99%IA/kg) of ^188^Re-liposome by nasopharyngeal carcinoma is not as high as that for a lung tumor (7.87%IA/kg) at 24 h after injection of ^188^Re-liposome, as shown in Additional file 1: Table S1. However, the uptake of ^188^Re-liposome is very low in the nasopharyngeal cavity so tumor targeting and localization was seen in the SPECT imaging for Patient 14 (Fig. 3b). The biodistribution data (Additional file 1: Table S1) shows a significant difference in the biodistribution of nano-targeted ^188^Re-liposomes in individual tumors for the 12 patients. In particular, the ratio of uptake for a tumor to a non-tumor site—adjacent soft tissue—at 24 h for patient 14 is 34.97.

The dosimetry study of the absorbed dose of radiation from the intravenous injection of ^188^Re-liposome (111 MBq) shows that the normal organs that absorb the highest doses are the spleen (1.38 ± 1.81 mGy/MBq), liver (0.92 ± 0.35 mGy/MBq), lungs (0.58 ± 0.28 mGy/MBq), red marrow (0.06 ± 0.01 mGy/MBq), and kidneys (0.32 ± 0.09 mGy/MBq), as shown in Table 4. The higher uptake in the spleen and liver might be due to the phospholipid in liposomes, which is removed by reticuloendothelial system (RES). The maximum tolerance dose (MTD) is defined as the dose that results in a 5% probability of complication within 5 years (TD 5/5). The reported respective MTDs for vital organs such as the liver, lungs, and red marrow are 25 Gy, 15 Gy, and 1.85 Gy [16, 45, 46]. After injection with 111 MBq ^188^Re-liposome, this study shows that the doses that are absorbed by the liver (0.10 Gy), lung (0.06 Gy), kidney (0.04 Gy), and red marrow (0.01 Gy) are much lower than the vital organ maximum tolerance doses. The clinical results for the doses that are absorbed by the vital organs for the 12 patients (Table 4) show the maximum respective dose of radioactivity that is delivered to the vital organs such as the liver, lungs, and red marrow due to the injection of dose of ^188^Re-liposome is 27.2 GBq (734 mCi), 25.9 GBq (699.3 mCi), and 32.5 GBq (876.3 mCi). The clinical results and dosimetry study show that the lungs and liver are dose-limiting critical organs, with an estimated maximum delivered radioactivity of 25.9 GBq and 27.2 GBq for ^188^Re-liposome radiotherapy for this subset of 12 patients. Using the results in Table 5, the maximum dose that is absorbed by lung tumors is from 5.36 Gy (patient 10) to 26.96 Gy (patient 14). The estimated maximum dose that is absorbed by liver tumor is from 23.3 Gy (patients 3 and 7) to 46.13 Gy (patient 12), which is much higher than the doses for phase 1 and the clinical dose-escalation trial study for hepatocellular and prostate carcinoma [22, 24]. However, treatment protocols that involve multiple cycles could reduce toxicity and improve the effectiveness of therapy. The total radioactivity of the injection and the doses that are absorbed by could also be increased.

The results for the 12 patients in the phase 0 study using a low injected activity (111 MBq) show that ^188^Re-liposome treatment does not result in any serious adverse events, as demonstrated by hematological, urinary, biochemical clinical data, dosimetric evaluation, and post-administrative follow-up. While some adverse events that are related to the study drug include chillness and palpitation during or right after injection, all of these adverse events are mild (grade 1 in CTCAE 4.03) and are resolved on the same day. Three SAEs were recorded, but none of these are related to ^188^Re-liposome. Two events were due to a scheduled chemotherapy session within 1 month after the injection of ^188^Re-liposome. Hematuria was also observed in patient 12 due to bladder metastasis, as confirmed by subsequent cystoscopic and pathological studies. It is worth noting that no subject discontinued the study due to AE/SAE. Two patients withdrew their consent prematurely due to an inability to tolerate the imaging procedure in one case and deteriorating liver function that was associated with metastases shortly after administration of ^188^Re-liposome in the other case. No clinically significant abnormalities in physical or laboratory examinations were recorded as adverse events that are related to ^188^Re-liposome.

A study by Harrington et al. found that no other adverse reactions were attributable to a 65–107 MBq In-111-liposome infusion and the repeat hematological and biochemical profiles performed at 10 days showed no significant changes [41]. In a phase 1 clinical trial of ^188^Re-lipiodol therapy for hepatocellular carcinoma in 11 patients, no clinical liver toxicity was recorded for administered doses of 1.86–4.14 GBq [22]. The experimental results for the laboratory parameters for this study, including hepatotoxicity, show no clinically significant abnormalities that are related to the injection of 111 MBq of ^188^Re-liposome. Table 5 lists the highest doses of ^188^Re-liposome that are absorbed in tumor lesions for 12 patients. Lung tumors absorb doses from 0.21 mGy/MBq (patient 10) to 1.04 mGy/MBq (patient 14) and liver tumors absorb 0.86 mGy/MBq (patient 3 and 7) to 1.70 mBq/MBq (patient 12).

The administration of ^188^Re-liposome (111 MBq) results in a therapeutic response in patient 11, who had esophageal cancer and lung metastases. This tumor absorbs a radiation dose of 35.1 mGy in lung, as shown in Fig. 4. Patient 14 has a nasopharyngeal tumor, whose primary tumor and lung metastases, respectively, absorbed a dose of 16.7 and 115.6 mGy. This nasopharyngeal tumor shows a clear response to low-radioactivity ^188^Re-liposome, as verified by nasopharyngoscopic examinations at 2 and 12 months (Fig. 3e, f) after injection.

The difference between the absorbed radioactivity and the unexpected tumor response could be due to different levels of radioactivity in the microenvironment of the tumor. The intratumoral penetration of liposome poses a great challenge for the effective delivery of therapeutics, especially for small molecules or biologics [47, 48]. For beta-emitting ^188^Re-liposome that is capable of delivering tumor-killing radioactivity at a range around 1–4 mm, the therapeutic need not be extensively distributed to achieve an ideal anti-cancer effect. The response may also be partially due to the effect of conventional treatments that these patients received before or after injection with ^188^Re-liposome, although they were administered at least 1 month apart in compliance with the protocol. Patient 11 had been taking oxalipatin, but stopped 1 month before enrollment in the ^188^Re-liposome trial. At that point, the tumor had progressed, as evidenced by the increasing size and number of lung metastases. The patient received no anti-cancer treatment after injection with ^188^Re-liposome until death, more than 11 months later. Patient 14 received cisplatin+gemcitabine after injection with ^188^Re-liposome so it is impossible to determine whether ^188^Re-liposome, follow-on chemotherapy, or both are responsible for the response of this tumor.

Since the publication of the results of the pivotal ipilimumab trial for metastatic melanoma in 2010, there has been significant development in immunotherapy, especially in relation to blockers of immune checkpoints, including CTLA-4, PD-1, and PD-L1, which have a durable beneficial effect in a proportion of cancer patients. There is also increasing evidence that the effect of radiation in terms of modulating immunity increases its potential role in systemic anti-cancer treatment, in combination with immunotherapy. Local irradiation of tumor-triggering systemic response is described as an abscopal effect, which was rarely noted before the introduction of immune checkpoint inhibitors (ICIs). However, in the past decade, many studies have demonstrated this effect and more than 100 clinical trials combining radiotherapy with ICIs or other immunotherapy (www.clinicaltrials.gov) have been documented. The vast majority of these clinical trials involve external beam radiotherapy with either conventional fractions or hyprofractional stereotactic body radiotherapy (SBRT). Therefore, only a limited number of tumors can be irradiated. However, ^188^Re-liposome can be systemically delivered and could potentially reach disseminated lesions. To determine the potential of the internal radiotherapeutics that are demonstrated by this study, the authors are currently conducting preclinical studies using ^188^Re-liposome in combination with ICIs, using syngeneic murine metastatic models.

Cancer patients with multiple metastases have very limited options so ^188^Re-liposome can be systemically delivered and could offer a means for the compassionate treatment of intractable or advanced tumors. Based on the biodistribution and dosimetry calculations for the doses (mGy/MBq) that are absorbed by normal organs, as shown in Tables 3 and 4, and the reported maximum tolerance dose (MTD) for vital organs such as the liver, lungs, and red marrow, the estimated respective limiting delivered dose of radioactivity from ^188^Re-liposome to the liver, lungs, and red marrow is 27.2 GBq (734 mCi), 25.9 GBq (699.3 mCi), and 32.5 GBq (876.3 mCi). The dose escalation trial protocol for phase 1 studies has been approved by the Taiwanese FDA. Six activity levels, ranging from 15.54 (0.42), 23.31 (0.63), 31.08 (0.84), 38.85 (1.05), 46.62 (1.26), and 54.39 (1.47) MBq/kg (mCi/kg), are planned and dose escalation proceeds sequentially between each dose. Each patient receives a therapeutic dose and is observed for 2 weeks before the next patient is recruited to the trial.

In summary, the encouraging results for the low radioactivity, nano-targeted radiotherapeutic, ^188^Re-liposome in this phase 0 clinical study justify a phase I dose-escalation clinical trial.

## Conclusion

The tumor to non-tumor radiotherapeutic-uptake ratios for the majority of the trial patients are acceptable. An interesting observation is that there is a tumor response in certain patients who receive low-dose radiation. This encouraging result warrants a phase I dose-escalation trial for patients with advanced and metastatic cancer.

## Abbreviations

%IA/kg: Percentage of injected activity per kilogram
ALP: Alkaline phosphatase
ALT: Alanine aminotransferase
AST: Aspartate aminotransferase
AUC: Area under the curve
BD: Biodistribution
CBC: Complete blood count
Cl: Total body clearance
CRFs: Case report forms
CTCAE: Common terminology criteria for adverse events
ECOG: Eastern cooperative oncology group
EPR: Enhanced permeability and retention
FDA: Food and drug administration
HEDP: Hydroxyethylidene diphosphonate
%IA/ml: injected activity per milliliter
LET: Linear energy transfer
*C*_max_: maximum concentration
MTD: Maximum tolerance dose
NOAEL: No observed adverse effect level
NPC: Nasopharyngeal tumor
NCA: Non-compartmental analysis
PK: Pharmacokinetics
PS: Performance status
RES: Reticuloendothelial system
SAE: Serious adverse event
SUV: Standardized uptake value
*T*_1/2z_: Terminal elimination half-life
ULN: Upper limit of normal
VOIs: Volumes of interest
